# Fasting plasma amino acid profiles after resistance exercise with or without nutritional supplementation in older adults: an exploratory analysis of secondary outcomes from a randomized controlled trial

**DOI:** 10.1016/j.jnha.2026.100948

**Published:** 2026-08-19

**Authors:** Koichiro Sumi, Satoshi Seino, Miki Narita, Yuri Yokoyama, Kinya Ashida, Shoji Shinkai

**Affiliations:** a Wellness Science Labs Meiji Holdings Co., Ltd. 1-29-1 Nanakuni, Hachiouji, Tokyo 192-0919, Japan; b R&D Division, Meiji Co., Ltd. 1-29-1 Nanakuni, Hachiouji, Tokyo 192-0919, Japan; c Research Team for Social Participation and Healthy Aging, Tokyo Metropolitan Institute for Geriatrics and Gerontology, 35-2 Sakae-cho, Itabashi-ku, Tokyo 173-0015, Japan; d Institute of Well-Being, Yamagata University, 2-2-2 Iida-nishi, Yamagata 990-9585, Japan; e Faculty of Human Welfare, Tokyo Online University, 1-7-3, Nishi-Shinjuku, Shinjuku, Tokyo, 160-0023, Japan; f Kagawa Nutrition University, 3-9-21, Chiyoda, Sakado, Saitama, 350-0288, Japan

**Keywords:** Older adults, Resistance exercise, Amino acids, Arginine, Glutamine, Supplementation, Randomized controlled trial

## Abstract

**Background:**

Resistance exercise and nutritional supplementation improve muscle health in older adults, but the metabolic significance of fasting plasma amino acid profiles remains unclear. This study examined changes in fasting plasma amino acid composition over a 12-week intervention period in which community-dwelling older adults underwent resistance exercise with or without protein plus micronutrient supplementation.

**Methods:**

Participants were assigned to exercise alone (Ex) or exercise plus protein and micronutrient supplementation (ExPM). Both groups completed the same supervised 12-week resistance exercise program, while the ExPM group additionally consumed protein- and micronutrient-fortified beverages daily. Fasting plasma amino acids were measured before and after intervention using LC–MS. Analyses were conducted primarily in the per-protocol set (n = 78; ExPM, n = 38; Ex, n = 40) using linear mixed-effects models adjusted for age and sex.

**Results:**

Total, essential, and non-essential amino acid concentrations generally decreased from baseline to post-intervention in both groups. Leucine and phenylalanine showed significant group-by-time interactions, with greater decreases in the Ex group than in the ExPM group. The global arginine bioavailability ratio (GABR; Arg/[Orn + Cit]) increased significantly in both groups, with larger observed increases among participants with lower baseline values. In contrast, glutamine-related ratios (e.g., Gln/TAA and Gln/[Glu + BCAA + Asp + Asn]) increased in the Ex group but remained largely unchanged in the ExPM group.

**Conclusions:**

Fasting plasma amino acid profiling revealed two temporal patterns during the 12-week intervention period: an increase in GABR observed under fasting conditions in both groups and nutrition-sensitive differences in glutamine-related ratios. These findings suggest that fasting plasma amino acid profiles may provide complementary metabolic insight into exercise–nutrition interventions in older adults.

## Highlights

• Fasting GABR increased over 12 weeks in both intervention groups.
• This increase was observed irrespective of supplementation status.
• Glutamine-related amino acid responses differed between groups.
• Fasting amino acid profiles may provide metabolic insight into exercise–nutrition interventions.

## 1 Introduction

Sarcopenia and age-related decline in skeletal muscle mass and function are major determinants of frailty, disability, and reduced quality of life in older adults [6,[13], [14], [15],^20^]. Resistance exercise is a key strategy to preserve muscle health, and combined exercise-nutrition approaches are often more effective than exercise alone, although the certainty and magnitude of benefit vary across studies [11,18]. The previously reported randomized controlled trial underlying the present work showed that low-dose dairy protein plus micronutrient supplementation during resistance exercise improved lean mass outcomes compared with exercise alone in community-dwelling older adults [11].

Although intervention studies usually focus on body composition and physical performance, emerging evidence suggests that fasting plasma free amino acid profiles may provide complementary information on whole-body amino acid handling, muscle-related phenotypes, and nutritional status in older adults under relatively steady-state conditions [2,22]. This concept is also supported by an intervention study in adults with metabolic syndrome, in which a 3-month diet and exercise program normalized plasma free amino acid profiles in parallel with metabolic improvement [19]. In the present trial, fasting plasma samples were collected before and after the 12-week intervention and analyzed by LC-MS, but these amino acid data were not included in the primary report [11].

Among amino acid-related indices, arginine bioavailability is of particular interest because arginine is the substrate for nitric oxide synthesis and competes with metabolic pathways involving ornithine and citrulline. The global arginine bioavailability ratio (GABR; Arg/[Orn + Cit]) has been used as an index of systemic arginine availability relative to catabolic flux and as a surrogate related to nitric oxide synthetic capacity [16,17]. More broadly, recent work also supports the relevance of arginine metabolism to human aging biology [9].

Glutamine-related indices may also be informative in this setting. Glutamine is a conditionally essential amino acid that plays central roles in nitrogen transport, amino acid exchange, and adaptation to catabolic or anabolic stress [2,3]. Recent studies in older adults have reported that circulating amino acid profiles, including glutamine/glutamate- and other muscle-related metabolic patterns, are associated with muscle-related phenotypes, possible sarcopenia, and nutritional status, supporting the relevance of fasting amino acid composition as a metabolic readout in aging populations [2,4,8,22].

Therefore, the aim of this exploratory analysis of secondary outcomes was to comprehensively examine changes in fasting plasma amino acid composition over a 12-week intervention period in older adults who underwent resistance exercise with or without protein plus micronutrient supplementation. As the primary trial [11] demonstrated functional benefits but limited lean mass gains with exercise alone, and the underlying mechanisms for the superior muscle accretion in the supplemented group remained unclear, we investigated whether fasting amino acid signatures could capture distinct metabolic patterns between the two intervention groups. Given the exploratory nature of this analysis, we did not test prespecified hypotheses for specific amino acids; instead, we profiled the overall amino acid responses to identify potential metabolic signatures related to the intervention context, ultimately highlighting arginine bioavailability and glutamine-related ratios as informative indices.

## 2 Materials and methods

### 2.1 Study design and participants

This study reports secondary outcomes (plasma amino acid data) that were collected as part of a randomized controlled trial, the primary outcomes of which have been published elsewhere [11].

Briefly, non-disabled men and women aged 65–80 years who were not engaged in regular exercise were recruited through community advertisements. Major exclusion criteria included regular participation in resistance exercise, abnormal clinical findings, serious chronic diseases, or dietary restrictions affecting protein intake. All participants provided written informed consent prior to participation. The study protocol was approved by the Ethical Committee of the Tokyo Metropolitan Institute of Gerontology and registered in the University Hospital Medical Information Network Clinical Trials Registry (UMIN-CTR; UMIN000015548).

### 2.2 Randomization and masking

After baseline assessments, participants were randomly assigned in a 1:1 ratio to either an exercise-alone group (Ex) or an exercise plus protein and micronutrient supplementation group (ExPM). Randomization was performed using computer-generated permuted blocks stratified by age and sex. Exercise trainers were masked to group allocation throughout the study. Due to the nature of the intervention, participant blinding was not feasible. However, outcome assessors were blinded to group allocation, and standardized procedures were used to minimize potential bias.

### 2.3 Intervention

Both groups participated in a supervised resistance exercise program twice weekly for 12 weeks. Each session consisted of warm-up, resistance exercises targeting major muscle groups using body weight, resistance bands, and light equipment, and cool-down. Exercise intensity was progressively increased and monitored using the OMNI Perceived Exertion Scale for Resistance Exercise (OMNI-RES) [10], targeting an intensity of approximately 5–7. In addition, participants were instructed to wear a pedometer throughout the study period and to record daily step counts in an exercise diary. They were encouraged to increase their daily steps in a phased manner (approximately +1000 steps per month). Detailed procedures and changes in physical activity during the intervention have been reported previously [11].

In addition to exercise, participants in the ExPM group consumed a milk protein–fortified beverage (providing approximately 10.5 g protein/day) and a micronutrient-fortified beverage containing vitamins and minerals (including vitamin D, B vitamins, and trace elements) daily throughout the intervention period. The detailed composition of the study beverages is presented in Supplementary Table S1 and has been described previously [11]. Participants in both groups were instructed not to change their habitual diet. Dietary intake was assessed using a 3-day food diary (2 weekdays and 1 weekend day), with detailed instructions provided to participants. Submitted records were reviewed by trained dietitians, and nutrient intake was calculated using a standardized software program. Detailed procedures and findings, including the stability of habitual dietary intake during the intervention, have been reported previously [11].

### 2.4 Secondary outcomes: plasma amino acid profiles

The present study reports an exploratory analysis of secondary plasma amino acid data that were collected as part of the original randomized controlled trial. Morning fasting blood samples were obtained from each participant’s antecubital vein between 8:30 and 10:30 AM after an overnight fast of at least 12 h at baseline and after the 12-week intervention. Baseline samples were collected on January 13 and 14, 2015, approximately 3 weeks before the start of the exercise program. The final supervised exercise session was conducted on April 24, 2015, and post-intervention samples were collected on April 27 and 28, 2015, corresponding to 3–4 days after the final exercise session.

For amino acid analysis, blood was collected into EDTA-Na tubes at the same sampling visit as the biochemical measurements reported in the parent trial. Samples were immediately cooled on ice, centrifuged under refrigerated conditions, and plasma was stored at −80 °C. Plasma samples were transported on dry ice to an independent laboratory (SRL, Inc., Tokyo, Japan) on the day of sampling and analyzed within approximately 1 week of sampling; samples were thawed only once, immediately before analysis. Plasma free amino acid concentrations were quantified by standardized liquid chromatography–mass spectrometry (LC–MS) procedures [12]. In addition to individual amino acids, composite indices including total amino acids (TAA), essential amino acids (EAA), branched-chain amino acids (BCAA), aromatic amino acids (AAA), the global arginine bioavailability ratio (GABR; Arg/[Orn + Cit]), and glutamine-related ratios were calculated.

### 2.5 Analysis population

The primary analyses of the present study were conducted in the per-protocol set, defined as participants who completed the intervention without major protocol deviations. The full analysis set, including all participants who completed post-intervention assessment, was used for sensitivity analyses. Of the randomized participants, 80 completed the intervention and post-intervention assessments. Two participants in the ExPM group were excluded from the per-protocol set due to poor compliance with supplementation, resulting in a per-protocol population of 78 participants: 38 in the ExPM group and 40 in the Ex group (Supplementary Figure S1).

### 2.6 Adherence and safety

Adherence to the exercise program was high in both groups (median attendance approximately 95%). Compliance with supplementation in the ExPM group was monitored using daily records. No serious adverse events were reported during the intervention. Mild gastrointestinal symptoms were observed in a small number of participants in the supplementation group and resolved without clinical consequences. Detailed participant flow and trial procedures are presented in Supplementary Figure S1 and have been described previously [11].

### 2.7 Statistical analysis

All values are presented as mean ± standard deviation. Changes in outcomes over time and between groups were assessed using linear mixed-effects models for repeated measures, with group, time, and their interaction as fixed effects, and participant as a random effect. Models included baseline-related covariates (e.g., age and sex) as appropriate. A compound symmetry covariance structure was used to account for within-subject correlations over time.

In this exploratory analysis, GABR and its related indices were considered the primary exploratory endpoints, while individual amino acids and other derived indices were treated as secondary or hypothesis-generating outcomes. Given the exploratory nature of the study and the number of outcomes examined, no formal adjustment for multiple comparisons was applied; therefore, statistical significance (P-values) for the secondary outcomes should be interpreted as hypothesis-generating. Statistical analyses were performed using SPSS Statistics version 29.0.1.0 (IBM Corp., Armonk, NY, USA). Graphs were generated using GraphPad Prism version 10 (GraphPad Software, San Diego, CA, USA).

## 3 Results

### Changes in individual plasma amino acids at the fasting state

Individual plasma amino acid concentrations at baseline and post-intervention are summarized in Table 1. Overall, total amino acids, essential amino acids, and non-essential amino acids decreased from baseline to post-intervention in both groups. Among essential amino acids, leucine and phenylalanine showed significant group-by-time interactions, with greater decreases in the Ex group than in the ExPM group. Among non-essential amino acids, arginine increased in both groups, whereas several amino acids including asparagine, alanine, glycine, glutamic acid, and proline decreased over time. Glutamine showed a trend toward a group-by-time interaction, with a decrease in ExPM and relative maintenance in Ex. Citrulline increased in both groups, with a greater increase in ExPM than in Ex, whereas ornithine decreased in both groups (Table 1).

**Table 1:** Concentrations of plasma amino acids at baseline and post-intervention between the ExPM and Ex groups^1^.

	ExPM group		Ex group		P-value^2^	
	Baseline	Post-intervention	Baseline	Post-intervention		
	Mean SD	Mean SD	Mean SD	Mean SD	Time effect	Group-by-time Interaction
EAA, nmol/mL						
Valine	199.5 ± 28.7	192.5 ± 25.8	207.9 ± 35.7	191.7 ± 30.2	**<0.001**	0.111
Leucine	107.9 ± 20.9	108.2 ± 14.2	113.2 ± 19	105.6 ± 14.9	**0.048**	**0.034**
Isoleucine	53.3 ± 9.7	48.8 ± 8.5	56.2 ± 11.8	49.2 ± 9.2	**<0.001**	0.218
Threonine	114.7 ± 22	102.7 ± 18.6	113.1 ± 23.1	107.1 ± 18.5	**<0.001**	0.195
Methionine	22.3 ± 2.9	23.1 ± 3.9	23.1 ± 4	22.6 ± 3.5	0.714	0.1
Phenylalanine	56.1 ± 5.7	57.1 ± 6.1	57.7 ± 5.7	54.8 ± 5.7	0.128	**0.002**
Lysine	182.9 ± 30.3	181.2 ± 23.7	175.8 ± 20.6	175.1 ± 19.7	0.681	0.856
Tryptophan	49.6 ± 8.7	48.4 ± 6.6	48.3 ± 8.6	46.4 ± 7.2	**0.016**	0.599
Histidine	74.9 ± 8.6	71.8 ± 6.2	77.7 ± 6.1	74.3 ± 6.8	**<0.001**	0.903
NEAA, nmol/mL						
Asparagine	44.4 ± 6.5	41.4 ± 5.8	44.5 ± 6	41.3 ± 4.6	**<0.001**	0.886
Aspartic acid^3^	3.9 ± 1.7	3.5 ± 1.5	3.9 ± 1.4	3.3 ± 1.4	**0.019**	0.551
Alanine	362.1 ± 66.5	333.4 ± 72.7	374.2 ± 78.9	328.9 ± 67.9	**<0.001**	0.248
Arginine	66.4 ± 24.8	83.9 ± 11.8	64.4 ± 26.3	81.9 ± 11.5	**<0.001**	0.989
Glycine	217 ± 56.1	207.4 ± 47.6	224.9 ± 68.7	213.8 ± 64.5	**0.002**	0.823
Glutamine	569.6 ± 53.7	547.3 ± 42.8	568.6 ± 58.3	567.5 ± 50.2	0.063	0.09
Glutamic acid	50.3 ± 19.5	39.6 ± 14	50.9 ± 18.3	35.1 ± 12.5	**<0.001**	0.167
Cystine	23.4 ± 6.6	33.8 ± 10	26.4 ± 8.2	31.8 ± 9.3	**<0.001**	**0.021**
Serine	109.1 ± 16.9	96 ± 16.1	108 ± 18.1	108 ± 18.6	**<0.001**	**<0.001**
Tyrosine	60.9 ± 9.9	60.5 ± 11.2	58 ± 9.7	56.1 ± 8.6	0.133	0.332
Proline	133.5 ± 43.2	124.2 ± 37.3	130.9 ± 33.6	119.8 ± 25.9	**<0.001**	0.695
Other AA, nmol/mL						
Citrulline	25.7 ± 6.2	30.5 ± 5.9	26.6 ± 5.1	27.9 ± 5.4	**<0.001**	**<0.001**
Ornithine	84 ± 36.8	57.6 ± 14.1	79.2 ± 30.2	51.8 ± 9.4	**<0.001**	0.88
Taurine	76.4 ± 18.3	79.5 ± 27.5	75.4 ± 15.4	72.1 ± 20.1	0.929	0.235
Σ Total AA, nmol/mL	2501.8 ± 227.3	2404.7 ± 201.4	2527.5 ± 243.4	2414.6 ± 192	**<0.001**	0.714
Σ NEAA, nmol/mL	1640.6 ± 156.6	1570.9 ± 146.7	1654.6 ± 174.7	1587.6 ± 131.2	**<0.001**	0.931
Σ EAA, nmol/mL	861.2 ± 103.1	833.8 ± 83.5	873 ± 101.4	827 ± 85.9	**<0.001**	0.3
Σ BCAA, nmol/mL	360.7 ± 56.1	349.5 ± 45.2	377.3 ± 63.2	346.5 ± 51.8	**<0.001**	**0.05**
Σ AAA, nmol/mL	117 ± 13.9	117.6 ± 15.7	115.6 ± 14.1	111 ± 12.8	0.071	**0.02**

1 Per-protocol set. All values are means ± SDs. ExPM = exercise plus protein and micronutrients; Ex = exercise alone; AA = amino acids; Total AA = total amino acids (EAA + NEAA); EAA = essential amino acids; NEAA = non-essential amino acids; BCAA = branched-chain amino acids; AAA = Aromatic amino acids; Fischer ratio = BCAA/AAA.

2 The significance of fixed effects for time and group-by-time interaction was assessed using repeated-measures mixed-effects models with sex and baseline age as covariates.

3 For four post-intervention values below the limit of quantification (one in the ExPM group and three in the Ex group), the corresponding pre-intervention value for each participant was imputed.

### Arginine-related indices

**Fig. 1:**
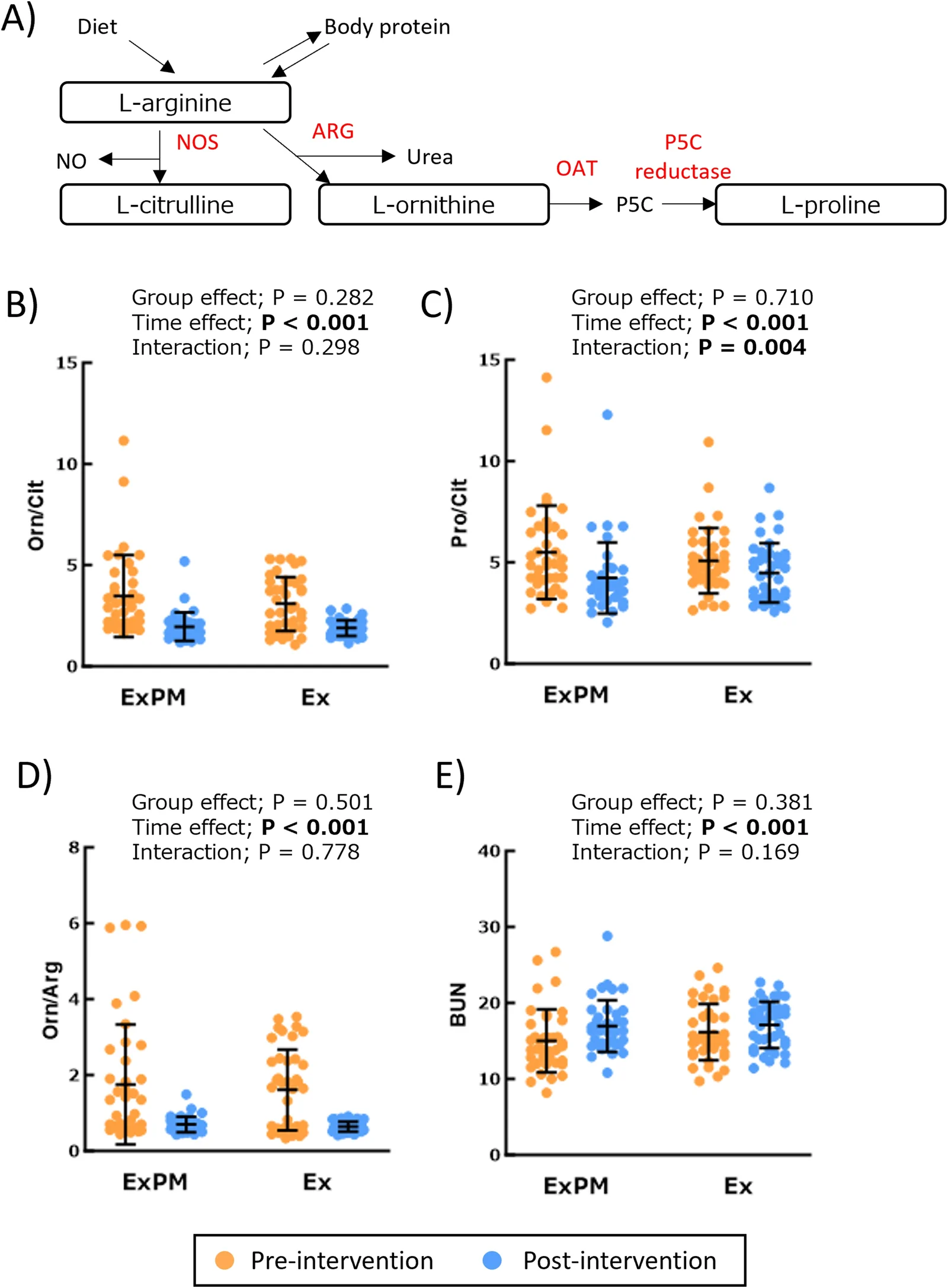
Relative changes in arginine metabolism-related indices at the fasting state. (A) Schematic overview of arginine metabolism. (B–E) Ratios or concentrations at baseline and post-intervention in the Ex and ExPM groups (per-protocol set). Orn/Cit and Orn/Arg decreased in both groups (time effect), Pro/Cit decreased more in ExPM than in Ex (group-by-time interaction), and blood urea nitrogen increased in both groups (time effect). Data are presented as means ± SD, with individual data points shown for reference. ARG, arginase; NOS, nitric oxide synthase; OAT, ornithine aminotransferase; P5C, pyrroline-5-carboxylate; Orn, ornithine; Cit, citrulline; Arg, arginine; Pro, proline.

Because several individual amino acids related to arginine metabolism changed after intervention, we next analyzed arginine-related indices and blood urea nitrogen. Orn/Cit and Orn/Arg decreased significantly over time in both groups, whereas Pro/Cit showed a significant group-by-time interaction, decreasing more in the ExPM group than in the Ex group. Blood urea nitrogen increased in both groups (Fig. 1).

**Fig. 2:**
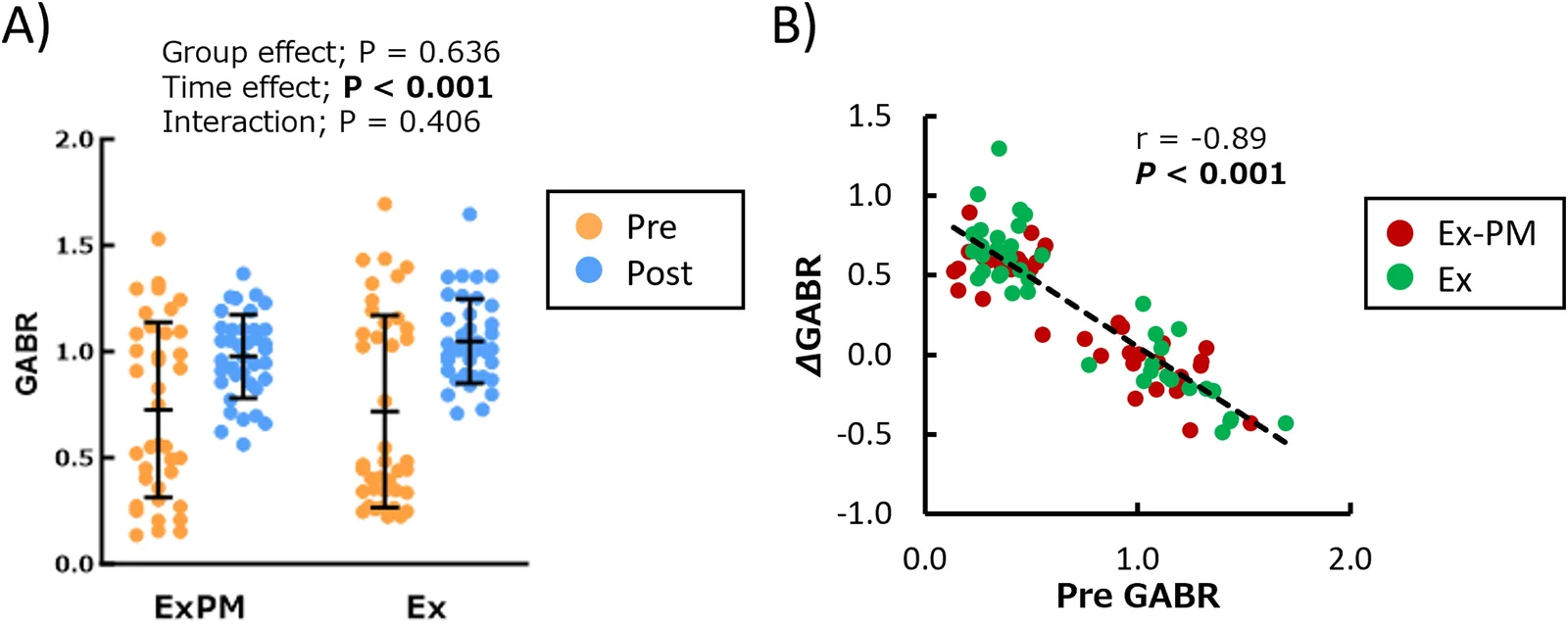
Global arginine bioavailability ratio (GABR) at fasting state. (A) GABR, defined as Arg/(Orn + Cit), at baseline and post-intervention in the Ex and ExPM groups. (B) Correlation between baseline GABR and change in GABR (ΔGABR). A strong inverse relationship was observed between baseline GABR and ΔGABR. Ex, exercise alone; ExPM, exercise plus protein and micronutrients. Data are presented as means ± SD, with individual data points shown for reference.

The GABR, defined as Arg/(Orn + Cit), increased significantly from baseline to post-intervention in both groups, with no significant group-by-time interaction (Fig. 2A). In addition, baseline GABR was strongly negatively correlated with the change in GABR (Fig. 2B), indicating a larger observed increase among participants with lower baseline values. To descriptively explore this baseline heterogeneity, participants were stratified according to baseline GABR (approximately <0.6 vs ≥0.6). As shown in Supplementary Table S2, the lower-GABR subgroup was slightly older and had higher fat mass and BMI, as well as modest differences in certain physical performance measures.

### Glutamine-related indices

**Fig. 3:**
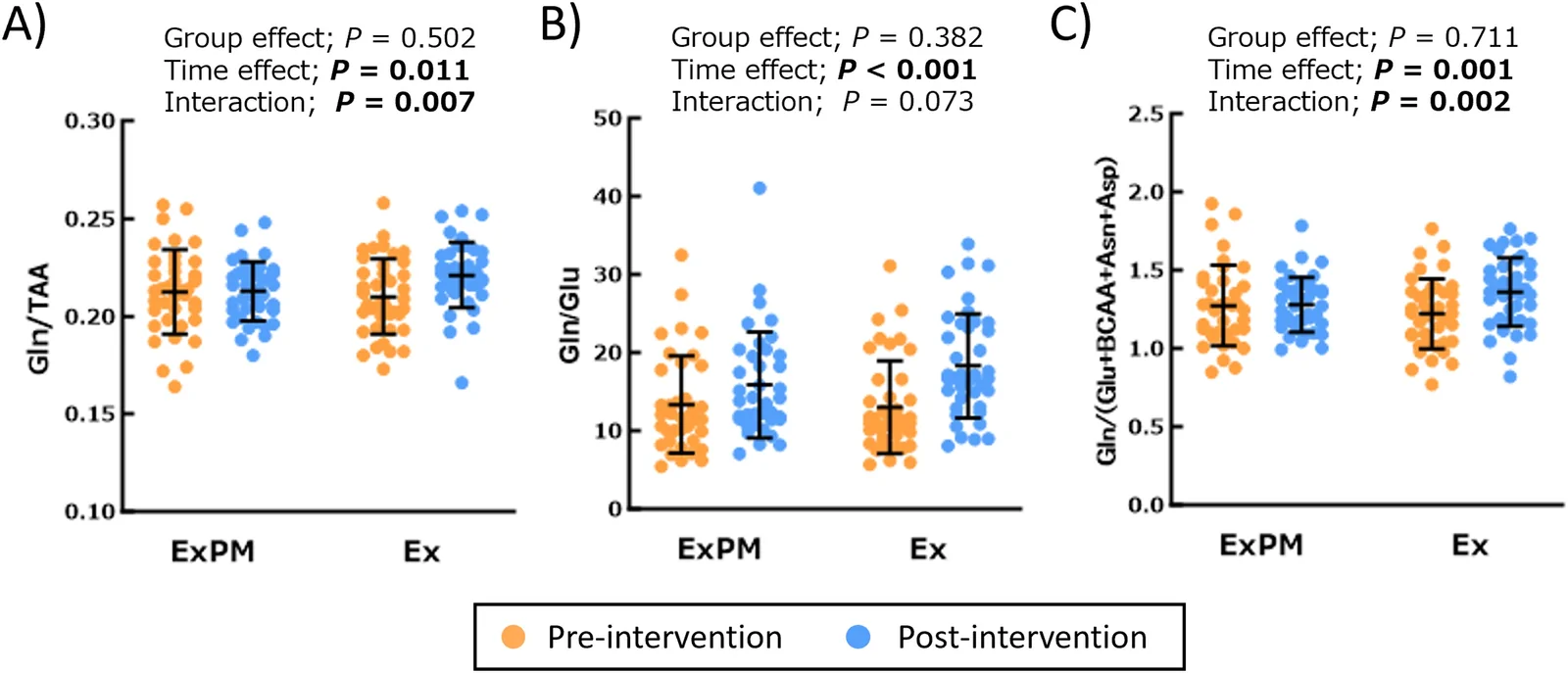
Relative changes in glutamine at fasting state. (A) Gln/TAA. (B) Gln/Glu. (C) Gln/(Glu + BCAA + Asp + Asn). These indices increased from baseline to post-intervention in the Ex group but showed little change in the ExPM group. Gln, glutamine; TAA, total amino acids; Glu, glutamic acid; BCAA, branched-chain amino acids; Asp, aspartic acid; Asn, asparagine. Data are presented as means ± SD, with individual data points shown for reference.

We then examined glutamine-related ratios to evaluate the relative behavior of glutamine within the fasting amino acid pool. Gln/TAA and Gln/(Glu + BCAA + Asp + Asn) showed significant group-by-time interactions, and Gln/Glu showed a similar directional trend. These indices increased from baseline to post-intervention in the Ex group but changed little in the ExPM group (Fig. 3).

## 4 Discussion

This study reports findings from an exploratory analysis of secondary outcomes from a 12-week randomized controlled trial in community-dwelling older adults [11], in which both groups underwent the same resistance exercise program and one group additionally received protein plus micronutrient supplementation. The present analysis identified temporal changes in fasting plasma amino acid profiles from baseline to post-intervention. The principal findings were twofold. First, the global arginine bioavailability ratio increased from baseline to post-intervention in both groups, without a significant group-by-time interaction, suggesting that higher fasting arginine bioavailability was observed during the 12-week intervention period, which included supervised resistance exercise in both groups, irrespective of supplementation status. Although the absence of a non-exercise control group precludes determination of whether this change was attributable specifically to resistance exercise, the finding raises the possibility that GABR may serve as an informative amino acid-related index in future exercise intervention studies in older adults. Second, glutamine-related ratios increased in the exercise-alone group but changed little in the supplemented group, indicating nutrition-sensitive differences in fasting amino acid responses during the 12-week intervention period. These findings are consistent with the patterns observed in Fig. 2, Fig. 3 and provide complementary metabolic information alongside the previously reported lean-mass advantage of the supplemented group [11].

GABR is commonly interpreted as an index of arginine availability relative to its downstream catabolism and has been used as a surrogate marker related to nitric oxide (NO) synthetic capacity in vivo [16,17]. In the present study, GABR was higher at post-intervention than at baseline in both groups, and the magnitude of change was similar between groups. In light of the shared exercise program in both groups, this finding should be interpreted as a descriptive temporal change rather than as evidence of an exercise-specific effect. Nevertheless, it suggests that GABR may be a useful amino acid-related index to consider in future studies of resistance exercise programs in older adults, given the close links of arginine availability and NO-related pathways with vascular and metabolic function and the age-related impairment of NO bioavailability [16,17].

An additional observation was that baseline GABR appeared to show a heterogeneous distribution, with larger increases from baseline to post-intervention among participants with lower baseline values. This pattern should be interpreted cautiously, because it may partly reflect baseline heterogeneity and regression to the mean. To explore baseline differences descriptively, participants were stratified according to baseline GABR. In this supplemental comparison, the lower-GABR subgroup was slightly older and had somewhat higher BMI and fat mass, as well as modestly poorer performance in some functional measures, including usual gait speed and 30-second chair stand performance (Supplementary Table S2). Because this subgrouping was not prespecified, these findings should be viewed as preliminary descriptive observations rather than evidence that GABR identifies clinically meaningful subgroups or predicts intervention responsiveness. Nevertheless, the observed pattern raises the possibility that baseline arginine-related amino acid status may provide a useful perspective for evaluating metabolic and functional heterogeneity in future exercise intervention studies in older adults.

This possibility may be relevant because GABR is used as a surrogate index related to arginine availability and NO metabolism [17], and reduced NO bioavailability has been reported in sedentary older adults, whereas lifelong physical activity appears to preserve NO bioavailability in the arterial circulation and skeletal muscle [7]. Moreover, NO has been implicated in vascular regulation, skeletal muscle function, and exercise physiology [5]. Thus, it would be valuable for future trials with prespecified analytical plans to examine whether baseline GABR and longitudinal changes in GABR are associated with improvements in gait speed, chair rise performance, handgrip strength, and other clinically meaningful outcomes, as well as vascular function or blood flow-related indices.

By contrast, glutamine-related indices showed a different pattern between groups. Ratios such as Gln/TAA and Gln/(Glu + BCAA + Asp + Asn) increased in the exercise-alone group but remained relatively unchanged in the supplemented group, indicating that concomitant nutritional support modified fasting amino acid responses during adaptation to exercise. Glutamine is a conditionally essential amino acid that plays central roles in nitrogen transport, amino acid exchange, and metabolic adaptation to physiological stress [2,3]. One possible interpretation is that the Ex group underwent the 12-week program with increased physical activity but without substantial changes in habitual dietary intake, as reported in the parent trial, whereas the ExPM group received additional protein and micronutrients. This difference in nutritional support may have contributed to the divergent fasting glutamine-related responses between groups. Given that the primary trial demonstrated greater gains in lean mass in the supplemented group [11], the absence of a glutamine-ratio increase in ExPM may be consistent with more favorable amino acid utilization during anabolic adaptation [1,18]. However, these interpretations remain speculative and should be regarded as hypothesis-generating.

The broader amino acid changes support the presence of nutrition-sensitive differences in fasting amino acid profiles. Total, essential, and non-essential amino acids generally decreased from baseline to post-intervention in both groups, whereas leucine and phenylalanine were relatively preserved in the supplemented group. This pattern may reflect, at least in part, the additional intake of protein and micronutrients in the ExPM group under conditions in which habitual dietary intake was largely maintained, as reported in the parent trial [11]. Because both groups underwent resistance exercise, the present design cannot isolate the effect of exercise itself; nevertheless, the group-by-time interactions observed for some amino acids suggest that daily protein and micronutrient support was associated with distinct fasting amino acid patterns during the intervention period. The relative preservation of leucine may be relevant to anabolic amino acid availability, but fasting BCAA concentrations should not be interpreted simply as favorable or unfavorable, because elevated circulating BCAAs have been linked to adverse metabolic states and recent work suggests that individual BCAAs, particularly isoleucine and valine, may differ in their metabolic implications [21]. Thus, the physiological meaning of the relative preservation of leucine and phenylalanine remains uncertain and should be examined in future studies in relation to metabolic health, muscle adaptation, and functional outcomes. Similar responsiveness of plasma free amino acid profiles to lifestyle interventions has been reported in other populations [19]. Taken together, the present findings suggest that fasting amino acid signatures may provide complementary metabolic insight beyond conventional body composition outcomes in exercise–nutrition interventions for older adults.

This study has several limitations. First, this was an exploratory analysis of secondary outcomes from a trial originally designed for other primary outcomes, and because no non-exercise control group was included, the present design does not allow us to determine whether the temporal changes observed in both groups were specifically attributable to the exercise intervention. Second, only fasting samples were analyzed, and no postprandial or acute post-exercise amino acid kinetics were available. Third, because the nutritional intervention combined dairy protein and micronutrients, their individual contributions cannot be separated. Fourth, the physiological interpretation of GABR and glutamine-related ratios remains indirect, as no direct measures of nitric oxide production, arginase activity, vascular function, or muscle protein turnover were obtained. Fifth, the exploratory associations between amino acid signatures and clinical phenotypes should be interpreted cautiously because the study was not powered or designed to test mediation or moderation of functional outcomes. In addition, participants were not blinded to the intervention; however, the primary outcomes of the present analysis were objective biochemical measurements, which are less susceptible to expectation bias.

In conclusion, fasting plasma amino acid profiling revealed two complementary temporal patterns during the 12-week intervention period in older adults: an increase in the global arginine bioavailability ratio observed under fasting conditions in both groups and a nutrition-sensitive divergence in glutamine-related amino acid balance. Fasting amino acid signatures may provide complementary metabolic insight into exercise–nutrition interventions for older adults and warrant further investigation in studies designed to link amino acid profiles with functional and vascular outcomes.

## Author contributions

Conceptualization, K.S. and K.A.; methodology, K.S., S. Seino, K.A., M. N., Y.Y., and S. Shinkai; formal analysis, K.S., and S. Seino; investigation, S. Seino, M.N., Y. Y. and S. Shinkai; resources, K.S. and K.A.; data curation, S. Seino; writing—original draft preparation, K.S.; writing—review and editing, S. Seino, M.N., Y.Y., K.A., and S. Shinkai; data visualization, K.S.; and supervision, S. Shinkai. All authors have read and agreed to the published version of the manuscript.

## Declaration of Generative AI and AI-assisted technologies in the writing process

During the preparation of this manuscript, the authors used ChatGPT to assist with English language editing and clarity of expression. The authors reviewed and edited the content as needed and took full responsibility for the final manuscript. Generative AI or AI-assisted technologies were not used to create or modify figures, images, or artwork.

## Funding sources

The original clinical trial was conducted under a research agreement between the Tokyo Metropolitan Institute of Gerontology and Meiji Co., Ltd., and was supported by Meiji Co., Ltd. Meiji Co., Ltd. provided the study products and support for the conduct of the original trial. No specific external funding was received for the present exploratory analysis of secondary outcomes.

## Data availability

The anonymized raw data that support the findings of this study are available from the corresponding author, K. Sumi, upon reasonable request.

The data that has been used is confidential.

Data will be made available on request.

## Declaration of competing interest

During the conduct of the original clinical trial, K.S. and K.A. were salaried employees of Meiji Co., Ltd., which provided funding, study materials, facilities, and staff support for the study. The contributions of all authors are described in the Author Contributions section. The remaining authors declare no competing interests.
