# Supplementary material for: Fasting plasma amino acid profiles after resistance exercise with or without nutritional supplementation in older adults: an exploratory analysis of secondary outcomes from a randomized controlled trial
Source: J Nutr Health Aging. 2026 Aug 19;30(10):100948. doi: 10.1016/j.jnha.2026.100948 (PMC13519640; doi:10.1016/j.jnha.2026.100948)
Supplement: Supplementary file 1 [file mmc1.docx]

| Component | Protein-fortified  milk-based beverage^1^ | Micronutrient beverage^2^ |
| --- | --- | --- |
| Energy, kcal | 114 | 45 |
| Protein, g | 10.5 | 0.7 |
| Fat, g | 3.9 | 0 |
| Carbohydrate, g | 9.3 | 10.5 |
| Vitamins |  |  |
| *β*-carotene, *μ*g | - | 7200 |
| Vitamin D, *μ*g (IU) | - | 5.0 (200) |
| Vitamin E, mg | - | 30 |
| Vitamin B_1_, mg | - | 3.0 |
| Vitamin B_2_, mg | - | 4.0 |
| Niacin, mgNE | - | 32 |
| Vitamin B_6_, mg | - | 6.0 |
| Vitamin B_12_, *μ*g | - | 12 |
| Folic acid, *μ*g | - | 200 |
| Pantothenic acid, mg | - | 12 |
| Vitamin C, mg | - | 320 |
| Minerals |  |  |
| Calcium, mg | 337 | 17^3^ |
| Iron, mg | - | 7.5 |
| Zinc, mg | - | 8.0 |
| Copper, mg | - | 0.40 |
| Selenium, *μ*g | - | 25 |

**Supplementary table S1.**

^1^Per 200-mL serving; 1 serving supplied 10.5 g of total milk protein.

^2^Per 125-mL serving.

^3^Representative value.

**Supplementary table S2.**

| Variable |  | GABR cluster (FAS) ^1^ | | | | | | |  | *P*-value^2^ |
| --- | --- | --- | --- | --- | --- | --- | --- | --- | --- | --- |
|  |  | Lower group  n = 43 | | |  | Higher group  n = 37 | | |  |  |
|  |  | Mean |  | SD |  | Mean |  | SD |  |  |
| Male, n (%) |  | 6 (14.0) | | |  | 7 (18.9) | | |  | 0.548 |
| Age |  | 74.5 | ± | 4.0 |  | 72.4 | ± | 4.3 |  | **0.031** |
| GABR |  | 0.354 | ± | 0.122 |  | 1.154 | ± | 0.210 |  | **< 0.001** |
| Body Composition |  |  |  |  |  |  |  |  |  |  |
| Weight, kg |  | 55.2 | ± | 5.9 |  | 52.9 | ± | 8.5 |  | 0.166 |
| Whole body FFM, kg |  | 37.8 | ± | 4.4 |  | 37.5 | ± | 6.8 |  | 0.809 |
| Whole body FM, kg |  | 16.1 | ± | 3.2 |  | 14.2 | ± | 3.5 |  | **0.011** |
| BMI, kg/m^2^ |  | 23.5 | ± | 2.6 |  | 22.2 | ± | 2.5 |  | **0.020** |
| SMI, kg/m^2^ |  | 7.00 | ± | 0.73 |  | 6.78 | ± | 0.94 |  | 0.255 |
| Physical performance |  |  |  |  |  |  |  |  |  |  |
| Hand-grip Strength, kg |  | 23.9 | ± | 5.7 |  | 23.0 | ± | 7.0 |  | 0.355 |
| One-leg standing with eyes open, s |  | 42.5 | ± | 21.9 |  | 47.0 | ± | 20.5 |  | 0.343 |
| Usual gait speed, m/s |  | 1.32 | ± | 0.17 |  | 1.41 | ± | 0.20 |  | **0.041** |
| Maximum gait speed, m/s |  | 1.76 | ± | 0.21 |  | 1.82 | ± | 0.21 |  | 0.156 |
| Timed Up-and-Go, s |  | 6.4 | ± | 1.2 |  | 5.9 | ± | 0.9 |  | 0.075 |
| 5-repetition chair stand, s |  | 8.1 | ± | 2.3 |  | 8.4 | ± | 1.4 |  | 0.603 |
| 30-second chair stand, n/30s |  | 20.3 | ± | 4.5 |  | 22.6 | ± | 4.3 |  | **0.020** |
| Knee-extension strength, Nm/kg |  | 59.4 | ± | 22.8 |  | 57.3 | ± | 24.4 |  | 0.695 |

^1^FAS=Full analysis set (Pre-intervention); GABR= global arginine bioavailability ratio;　Participants were stratified according to baseline GABR, with an approximate cut-off value of 0.6.

^2^ *P* values resulted in t-test except to sex ratio. *P* value of male ratio was tested by chi-square test.

FFM=Fat free mass, FM=Fat mass, BMI=Body mass index, SMI=skeletal muscle mass index

**Supplementary figure S1.**

ExPM = resistance exercise plus low-dose dairy protein and micronutrients

Ex = resistance exercise alone

PPS = per-protocol set
